# Accelerated Ageing Procedures to Assess the Stability of an Unconventional Acrylic-Wax Polymeric Emulsion for Contemporary Art

**DOI:** 10.3390/polym12091925

**Published:** 2020-08-26

**Authors:** Francesca Caterina Izzo, Eleonora Balliana, Emanuela Perra, Elisabetta Zendri

**Affiliations:** Sciences and Technologies for the Conservation of Cultural Heritage, Department of Environmental Sciences, Informatics and Statistics, Ca’ Foscari University of Venice, Via Torino 155/b, 30172 Venice, Italy; fra.izzo@unive.it (F.C.I.); 839127@stud.unive.it (E.P.)

**Keywords:** contemporary art materials, acrylic dispersion, acrilyc polymers, wax emulsion, accelerated ageing, binding media, Edelwachs Pigrol, conservation science, conservation

## Abstract

This research evaluates the stability of an aqueous emulsion of acrylic copolymers and waxes. Edelwachs, generally applied on wood, has been recently used as an unconventional medium in contemporary painting. Through Pyrolysis–Gas Chromatography–Mass Spectrometry (Py-GC-MS) and Fourier Transformed Infrared Attenuated Total Reflectance (FT-IR-ATR) analyses, the composition of Edelwachs was defined as a mixture of acrylic polymers (MA, MMA, nBA, nBMA), Carnauba and microcrystalline waxes and additives. Mock-ups-obtained mixing Edelwachs with titanium white, zinc white and ultramarine blue were subjected to UV, high temperatures, and high relative humidity accelerated ageing. The effect of the ageing procedures was evaluated through optical microscopy, colourimetric measurements, FT-IR-ATR, Thermogravimetry and Differential Scanning Calorimetry (TG-DSC) and wettability measures. FT-IR-ATR spectra do not show significant variations in terms of chemical stability, indicating a fair stability of Edelwachs as a painting binder. UV and high temperature treatments show the most relevant effects in terms of colorimetric changes (increasing of b*) and thermal stability. The TG-DSC highlights the influence of the pigments (specifically zinc white) mainly on the thermal behaviour of the acrylates. The unexpected decrease of wettability of the paint films, registered after ageing, may indicate a possible phase separation among acrylates and waxes.

## 1. Introduction

In the last decades, the scientific literature has been enriching in research related to the study and the stability of polymeric films in the field of Heritage and Conservation Science for contemporary art. This topic represents a relevant and current focus, also in consideration of the relative instability, sometimes unexpected, found in numerous artworks. The assessment of the ageing and durability of these polymers is, thus, fundamental for studying and understanding their behaviour over time and for preserving artworks in which they have been (sometimes unconventionally) employed as binding media and coatings or finishing layers.

This study takes into consideration an anomalous pictorial binder used in contemporary art, never studied either from a compositional point of view or regarding its stability over time; this product, named Edelwachs, is commercially sold as a wood coating produced by Pigrol (Tikkurila GmbH, Germany). According to the technical sheet, it consists of a complex mixture containing wax emulsion, acrylic dispersion, additives, organic and mineral pigments and water. 

The iconographer Fabio Nones (Trento, 1961) has been experimenting with its use both as a varnish and a painting medium from the early 1990s, developing his artistic technique based on it. According to the artist, the reduced drying times, and the compatibility with different supports (wood, canvas, parchment, paper and marble) make the product an interesting and good alternative to the traditional egg tempera in the production of religious icons. He also reports that his artworks created using Edelwachs mixed with inorganic pigments have not shown any relevant degradation signs in the last 30 years (personal communication from the artist). Nowadays, the use of Edelwachs, as a painting binder, has become common also among many of Nones’ students and pupils in Italy and is widespread across Europe. 

Hence the need to know and better understand this product, which has accidentally arrived in the world of artistic materials. The main aim of this paper was to study the stability and the durability of paint films obtained with Edelwachs and mixed with three different inorganic pigments, namely ultramarine blue, titanium and zinc white. 

The choice of the pigments was made according to the artist and considering that they are among the pigments that are most used even if mixed and are known for their influence on the stability of the pictorial film.

While for the ultramarine blue and the titanium white there are many studies in the literature regarding their relationship with the stability of acrylic [1,2,3,4,5,6], little exists regarding the white of Zn as an acrylic based emulsion pigment. Many of the publications refer either to the zinc–oil mix or to the antimicrobial properties of the zinc nano-particles [4,5,6,7,8,9].

The current research was developed in different steps: (i) Characterisation of the starting materials; (ii) assessment of morphological and chemical–physical variations before and after natural and accelerated ageing procedures (by high temperature, UV and high relative humidity exposure); (iii) assessment of the thermal behavior.

The obtained results have been compared with data from studies dealing with more conventional polymers used in contemporary art. In this sense and considering the presumable composition of Edelwachs, the focus was on waxy and acrylic compounds.

While for natural and synthetic waxes emulsions there is not much scientific literature regarding their use as pictorial binders, acrylic and methacrylic polymers have been and are extensively used in the formulations of modern and contemporary paints and surface coatings [10,11,12,13]. The stability of acrylic-based paint films has been tested through ageing procedures, for instance by exposure to UV light, high temperature and different percentage of Relative Humidity (RH). As reported in the literature [1,3,9,14,15,16], the exposure to UV light may produce chain scission and cross-linking reaction and the formation of decomposition compounds such as hydroperoxides, alcohols, ketones, anhydrides and γ-lactones. The exposure of acrylic paints to high temperature affects the alkyd side group; additionally, the scission of the alkyd groups seems to prevail over the cross-linking reaction [15]. High % of RH are reported to be potentially critical for acrylates [17] because of the water interaction with the water-sensible compounds present in the formulations, such as surfactants [4]. Literature studies highlight, in fact, the influence of additives in the stability of the paint films, both in terms of visual appearance and chemical–physical and mechanical behaviour [4,18,19].

Besides, it is also necessary to consider the influence of pigments on the stability of the overall paint systems. In particular, it has been observed that some pigments can have a protective stabilising effect in the paint film, while others can catalyse and accelerate the degradation of the polymer fraction [1,20].

From these studies, the importance of facing the complexity of the pictorial systems and the need to evaluate their behaviour to develop specific preventive actions essential for the conservation of contemporary works of art is clear. 

## 2. Materials and Methods 

### 2.1. Samples Preparation

For evaluating the stability of the unpigmented and pigmented films, Edelwachs, distributed by Pigrol (Tikkurila GmbH, Germany), was mixed with titanium white Ti0_2_ (C.I. PW6), zinc white ZnO (C.I. PW4) and ultramarine blue Na_8_-10Al_6_Si_6_O_29_S_2-4_ (C.I. PB29). 

Titanium white was purchased by Bresciani (Bresciani S.r.l., Milan, Italy), while ultramarine blue and zinc white were sold by Abralux Colori Berghè (Abralux Colori Beghè, Como, Italy).

Prior to casting, all pigments were finely powdered in an agate mortar. Pictorial mock-ups were obtained by mixing the pure binder with each pigment and casting the mix directly on glass slides for a total of 12 replicates for each mixture and the pure binder. To obtain a buttery consistency of the paints and in accordance also with the artist’s procedure, different pigment volume concentrations (PVC) were employed, as reported in Table 1. The thickness of the dried films varied approximately from 190 μm to 260 μm. The dry film thickness was measured using a calliper subtracting the slide glass contribution [21].

After casting, the paints were kept in a horizontal position and left to dry in room conditions (20 ± 2 °C, 55 ± 3 RH%). After that, they were stored under controlled humidity in a desiccator until a constant weight was reached. This was reached after 30 days and was considered as the starting point for further evaluation of morphological, chemical–physical and thermal changes.

### 2.2. Natural and Accelerated Ageing Procedures

A set of three replicates of unpigmented and pigmented films were subjected to four different ageing procedures selected and adapted based on the field literature [1,22,23,24]:

- Natural Ageing (NA): In laboratory conditions, at 20 ± 2 °C and 55 ± 3% relative humidity for a total ageing time of 1488 h;
- High Temperatures (HT) at 100 °C and 55% relative humidity for a total ageing time of 1488 h;
- High Relative Humidity at 85% (RH) was achieved using a glass desiccator containing a saturated solution of KCl salt [25,26], 20–22 °C, for a total ageing time of 1488 h;
- Photo oxidation by exposure (UV) to an OSRAM Ultra-Vitalux^®^ solar lamp (300 W, 230 V) in a ventilated chamber (45% RH; 31–32 °C), for a total ageing time of 1488 h. The wavelength of the light emitted was from 280 to 2000 nm (13.6 W in the range 315–400 nm, 3.0 W in the range 280–315).

### 2.3. Analytical Methods

#### 2.3.1. Characterisation of Starting Materials

Before the mock-ups production, Edelwachs and pigments were fully characterized to assess their chemical composition.

The elemental composition of the powdered pigments was determined using a Philips MiniPal X-ray fluorescence spectrometer with a rhodium cathode and operating at 20–40 keV (PANalytical B.V., Almelo, The Netherland). The identification of elements was obtained using Minipal software (PANalytical B.V., Almelo, The Netherland) by comparing literature data and data previously collected by the authors [27]. 

Fourier Transformed Infrared analyses in Attenuated Total Reflectance mode (FT-IR-ATR, Thermo Fisher Scientific Spa, Rodano, Italy) were performed on powdered pigments and pure Edelwachs with a Thermo Nicolet Nexus 670 FTIR spectrophotometer combined with a Smart Orbit Single Reflection Diamond ATR accessory, from 4000 to 400 cm^−1^ for 128 scans with 4 cm^−1^ resolution. Spectra were elaborated with Omnic 10.0 and Origin 9.0 software (OriginLab Corporation, Northampton, Massachusetts, USA).

Edelwachs emulsion was analysed using Thermally Assisted Hydrolysis and Methylation Gas Chromatography–Mass Spectrometry (THM–GC–MS) in combination with pyrolysis. The emulsion was treated with a few drops of a 2.5% solution of tetra methyl ammonium hydroxide (TMAH) in methanol and transferred to a pyrolysis eco-cup. The pyrolysis temperature was 550 °C. A 3030D Frontier pyrolyser (Frontier Lab, Fukuschima, Swizterland) was used, coupled with a Thermo Scientific Focus GC/ISQ mass spectrometer (Thermo Fisher Scientific Spa, Rodano, Italy). The separation was achieved through an HP5 column with a length of 30 m, an internal diameter of 0.25 mm and a film thickness of 0.25 μm. Helium (SIAD, Venezia, Italy) was used as carrier gas with a constant flow of 1 mL/min. The temperature program set was 35 °C (1 min)–60 °C/min–110 °C–14 °C/min–240 °C–5 °C/min–315 °C (2 min). The column was directly coupled to the ion source of the mass spectrometer. The temperature of the interface was 240 °C, the temperature of the ion source was 220 °C. Mass spectra were recorded from 20 until 600 amu, 7 scans per second. 

Xcalibur 2.1 software (Thermo Fisher Scientific Spa, Rodano, Italy) was used for collecting and processing mass spectral data. The pyrograms acquired were compared with a reference database of natural and synthetic materials, both available in the literature and ad-hoc created by the authors [28,29,30,31]. Interpretation of the GCMS results was made using the ESCAPE system, an expert system for characterizing py-GCMS data using AMDIS & Excel, created and developed by van Keulen and Schilling [32].

#### 2.3.2. Assessment of Morphological and Chemical–Physical Variations after Natural and Accelerated Ageing Procedures

All naturally and artificially aged mock-ups were investigated to assess their stability in terms of morphological and chemical–physical variations. 

The mock-ups were observed using an Olympus SZX16 optical microscope (Olympus, Tokyo, Japan) equipped for microphotography with a Camedia Olympus C-5050 (Olympus, Tokyo, Japan) to evaluate morphological variations due to natural and accelerated ageing.

The wettability of the mock-ups was obtained by contact angle measurements with the sessile water drop method according to the standard NorMaL 33/89 [33]. Ten measurements were performed for each mock-up and the average value was calculated. 

Colorimetric measurements were collected with a portable spectrophotometer (Konica Minolta CM-700d, Tokyo, Japan) equipped with CM-S100w (SpectraMagicTM NX, Tokyo, Japan) software. Measurement conditions were as follows: Illuminant D65, observer 8 degree viewing angle geometry and a 3 mm diameter target area. The colorimetric parameters were analysed by considering the CIELAB colour system (CIE, 1986), which represents each colour as Cartesian coordinates. L a b (CIELab-space) were recorded in SCI (Specular Component Included) modality. The total colour difference ΔE* was calculated according to the equation:

(1) $${\Delta E}^{*}=\;\sqrt[{2}]{{\Delta L}^{*2}+{\;\Delta a}^{*2}+{\Delta b}^{*2}}$$

where ΔL* = L(tx) − L(t0); Δa* = a(tx) − a(t0); Δb* = b(tx) − b(t0) are the differences calculated on the aged paint films (tx) with respect to the original (t0) paint layer. For each determination of L*a*b* values the mean value and standard deviation of three measurements were calculated. The measuring head (3 mm diameter) was placed, with the aid of a positioning mask, on the same area of glass slide film samples before and after the accelerated ageing. In the field of Heritage Science, generally, ΔE values higher than 5 are not acceptable since the total colour variation is clearly detectable [34,35,36].

Spectroscopic analysis through FT-IR-ATR (see Section 2.3.1—“Characterisation of Starting Materials”—for technical details) were performed at the beginning and the end of each ageing procedure on all the mock-ups. 

The obtained spectra were compared by using, as a reference, the peak at 1385 cm^−1^ (related to out of the plane CH bending). The difference in the IR spectra was useful to identify variations in the intensity of signals following the ageing procedures for unpigmented and pigmented mock-ups.

#### 2.3.3. Assessment of the Thermal Behaviour before and after Ageing Procedures

The thermal behaviour of selected mock-ups after and before ageing was studied using thermogravimetry (TG) and Differential Scanning Calorimetry (DSC) (NETZSCH-Gerätebau GmbH, Selb, Germany). TG and DSC analysis were performed simultaneously using a Netzsch 409/C apparatus. Thermal analyses were carried out at a heating rate of 10 °C/min 20 °C to 1000 °C. The instrument was purged with a mixture of air and N_2_ at a flow rate of 40 mL/min. The sample weight ranged between 0.5 and 2 mg; samples were collected with the help of a scalpel from the glass slides and put in an aluminium crucible. Alumina was used for internal calibration. Three replicates were performed for each sample. Data were collected with STA Netzsch software and then elaborated with Origin 9 software (OriginLab Corporation, Northampton, Massachusetts, USA) [3,37,38,39,40,41,42,43].

## 3. Results and Discussion

### 3.1. Characterisation of Starting Materials

The results obtained by XRF and FT-IR-ATR analysis on the powdered pigments are reported in Table 2. Despite the regular occurrence of fillers, extenders and impurities in the industrial production of painting materials, the composition of the selected pigments shows a high degree of purity. 

This finding is fundamental to better understand the influence of pigments in the stability of the painted films.

The composition of Edelwachs was investigated by means of FTIR-ATR and Py-GC-MS. 

According to the technical datasheet, Edelwachs is a complex mixture containing wax emulsion, acrylic dispersion, additives, organic and mineral pigments and water.

**Figure 1:**
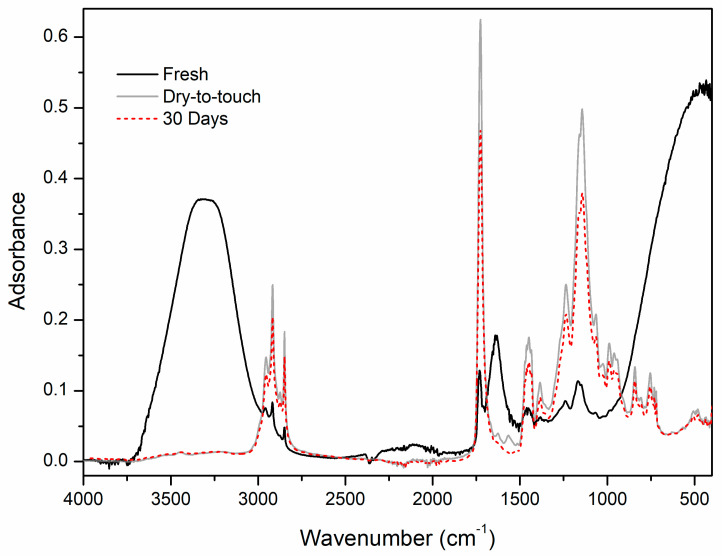
FT-IR-ATR spectra of fresh, dry-to-touch film and 30 days Edelwachs films.

Figure 1 depicts the FT-IR-ATR spectral profiles of fresh, dry-to-touch and 30-day-old film obtained from Edelwachs. 

In the fresh Edelwachs, the bands related to the stretching and the bending of the -OH in the emulsion are strongly visible at 3319 and 1641 cm^−1^, respectively.

Following the formation of the film, the peaks related to the absorption of the hydroxyl groups are no longer present. Besides, several absorptions from the Edelwachs binding media are more intensely visible [44,45,46]:

- The narrow and sharp peaks at 2954 cm^−1^ and 2869 cm^−1^ are associated with the asymmetric and symmetrical stretching of the methyl -CH_3_ group, respectively, due both to the acrylic dispersion and the wax emulsion;
- Similarly, the narrow and sharp absorptions at 2917 and 2849 cm^−1^ related to the asymmetric and symmetric stretching of the methylene group are linked to acrylic copolymers and wax;
- The C=O stretching band at 1726 cm^−1^ and C-O-C stretching band at 1144 cm^−1^ of the ester absorptions are linked to the presence of the acrylic emulsion; they may also indicate the co-presence of a natural wax in the formulation;
- The peaks at 1450 cm^−1^ and 1461 cm^−1^ can be associated with -CH_2_ bending vibration, together with 1385 cm^−1^, which refers to out-plane CH bending;
- The absorptions at 1240 and 1167 cm^−1^ refer to the stretching of the group C-O and C-C;
- Two sharp rocking vibrations at 734 and 723 cm^−1^ of methylene groups characteristic of a compound with a long aliphatic chain, such as a wax;

Furthermore, in the dry Edelwachs it was possible to identify several peaks related to additives with unsaturations at 1623 cm^−1^ and surfactants at 1564 cm^−1^, most likely metal carboxylates added as dispersing agents [16].

It is interesting to highlight that, in the 30-day old film, the latter absorptions are no longer detected. This may indicate that, after a month at 20 ± 2 °C, 55 ± 3 RH%, these additives probably move from the surface into the paint films as reported also in literature [47]. 

**Figure 2:**
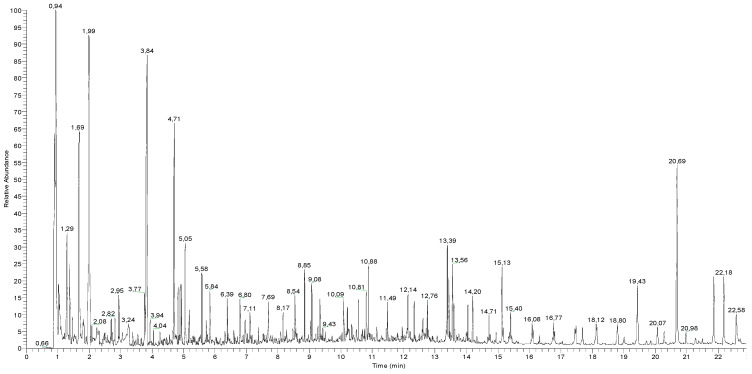
Total ion current pyrogram of Edelwachs after transesterification and Py-GC-MS analysis.

The results obtained by Py-GC-MS analysis (Figure 2 and Table 3) corroborated the IR findings and added more details about the complex organic fraction. 

**Figure 3:**
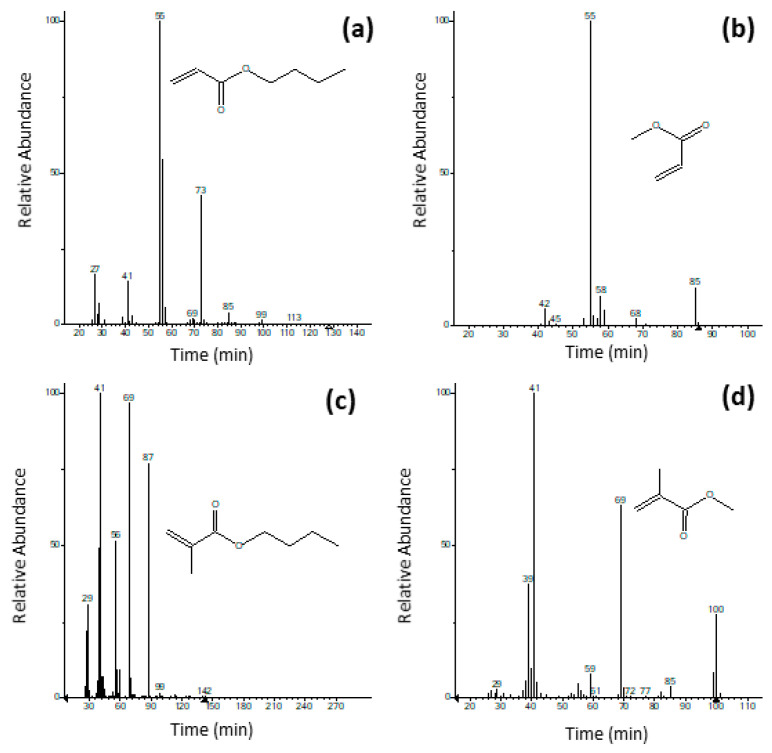
M/z spectra of (**a**) *n*-butyl acrylate, (**b**) methyl acrylate, (**c**) *n*-butyl methacrylate, (**d**) methyl methacrylate.

The acrylic dispersion Edelwachs is based on a co-polymer of methacrylate (MA), methyl methacrylate (MMA), *n*-butyl acrylate (nBA) and *n*-butyl methacrylate (nBMA) monomers, whose mass spectra are reported in Figure 3. Besides the main monomers, the pyrogram is also characterised by the presence of different oligomers (sesquimers, dimers, and trimers) at higher retention time [3,48,49], such as the trymer *n*-butyl acrylate at retention time (RT) of 15.13 min, which are commonly formed upon pyrolysis conditions. 

The wax emulsion turned out to contain both natural and synthetic waxy components. 

The core alcohol of high molecular weight in Carnauba (*Copernicia cerifera* Martius) wax, a palm tree that grows in Argentina, Brazil and Paraguay, is 1-Dotriacontanol, C32-OH, detected at RT 19.43 and 22.58 min [48,49]. Because of the presence of many *n*-alkanes, cycloalkanes and branched alkanes, as reported in Table 2, the waxy component appears to also have a microcrystalline nature [50,51,52,53].

Several additives were identified through Py-GC-MS. Cetyl alcohol, also known as 1-hexadecanol or palmityl alcohol, was identified at 8.54 min: This fatty alcohol, an end-product of the petroleum industry, or produced from vegetable oils, is commonly used as a paints and coatings additive (opacifier, emulsifier, and thickening agent). The long unsaturated compound 17-Pentatriacontene was found as well; it can be added in the formulation to improve gloss retention properties of wax-based coatings.

Many carboxylic acids, such as hexadecanoic acid, octadecanoic acid, octacosanoic acid and eicosanoic acid, were identified. They may be present in carnauba wax (with octacosanoic acid being the main one) and/or added as fluidifies in the commercial formulation. These carboxylic acids may also refer to the presence of metal carboxylates added as dispersing agents (e.g., metal stearates), as found in the IR spectrum of the dry Edelwachs film as well.

### 3.2. Edelwachs and Mock-Ups Post Treatments

According to literature related to weight changes in acrylic dispersion [10,54] and preliminary weight measurements, it was noted that the mock-ups reached constant weight within 30 days from the paint application. Therefore, the “time 0” (t0) is considered after 30 days from the paint application.

In the following sections, the results about the evaluation of morphological and chemical–physical variations occurring after accelerated ageing procedures and the thermal behaviour of the mock-ups are presented and discussed.

#### 3.2.1. Morphological and Colorimetric Changes

Any variation of the visual appearance of the painted surfaces to temperature, UV and RH may be linked to a change in the properties of the materials and therefore the evaluation of morphological and colorimetric changes is fundamental to assess the durability of paint films and their conservation. 

**Figure 4:**
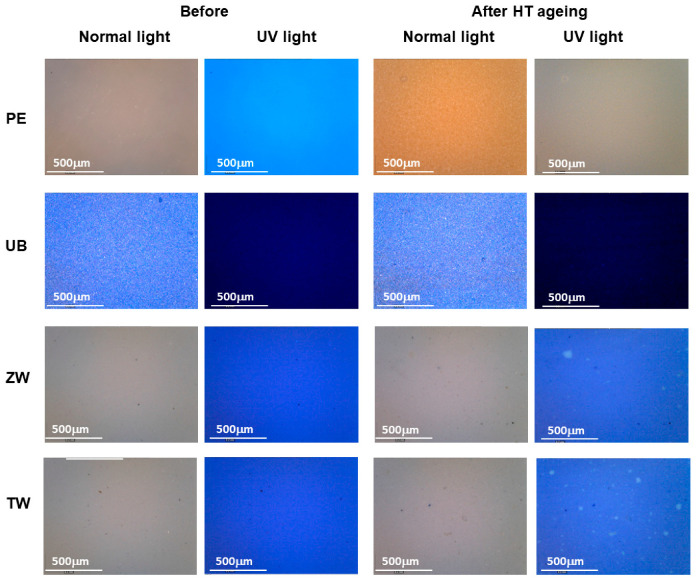
Microscopic observation of the mock-ups in normal and UV light before and after high temperature treatment: Unpigmented Edelwachs (PE), ultramarine blue (UB), zinc white (ZW) and titanium white (TW).

The variations in terms of macroscopic properties and colour parameters, after the selected different ageing procedures, can be summarised as follows:

- Natural Ageing: The mock-ups left at laboratory conditions did not show any substantial macro and micro-morphological variations. An exception is by the mock-up containing zinc white, which appeared slightly more opaque compared to the un-aged one. As concerns the colorimetric measurements, the variation in terms of L*, a*, b* was lower than 5, since the values of the ΔE are less than 1 for pigmented mock-ups and 3 for the Edelwachs film (Table 4), mainly due to the negative variation of the b* parameter.
- High Temperature: Microscopic observations of the surfaces of titanium and zinc white evidenced the formation of translucent spots, as depicted in Figure 4. These spots, which appeared to have a yellow-greenish fluorescence under UV-light observation (Figure 4), can be attributed to the migration of the surfactants from the paint bulk to the film surface. As seen before, FT-IR-ATR and Py-GC-MS detected the presence of several additives, among them surfactants. As widely discussed in the literature, accelerated ageing treatments (also with high temperature) can promote the migration of surfactants, which are then visible on the painted surfaces [47].

Nevertheless, these spots may be ascribable to the presence of natural and synthetic waxy components in the Edelwachs formulations: These compounds, having a melting point between 80 and 90 °C, may have moved from the film bulk after a phase separation from the acrylic dispersion. As reported in the literature, when waxes are present in water-based emulsions, the waxes can migrate to the surface, forming a thin re-solidified wax-layer enhancing the water repellency of the film [55,56]. 

In the case of the mock-up containing zinc white, another hypothesis can be made: The pigment is known to react with (free) fatty acid to form zinc carboxylates. Fatty acids were identified by Py-GC-MS, likely present in Carnauba wax and/or as rheological additives [57]. 

Differently from the white pigmented films, unpigmented Edelwachs and ultramarine blue mock-ups did not show any peculiar morphological variations.

**Figure 5:**
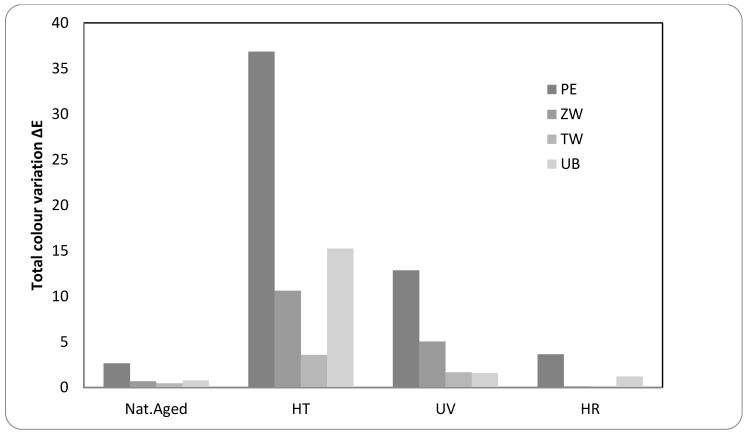
Total colour variations calculated as ΔE for all the mock-ups studied.

Total colour changes ΔE were significant for three mock-ups: The unpigmented film reached a ΔE value of 37, while zinc white was 11 and for ultramarine blue 15 (Figure 5). These values exceeded the value of 5, often considered as the limit value for colorimetric variation in Cultural Heritage. 

These colour changes turned out to be due to a very evident yellowing (increasing of b* parameter), followed by discoloration (decrease of L* parameter). As reported in Table 4, the measured Δb* values were +31, +9 and +12 for Edelwachs, zinc white and ultramarine mock-ups, respectively. The luminosity variation was very evident for the unpigmented mock-up, where an increase of the a* parameter was recorded as well. 

Literature generally reports that acrylic-based products undergo a fast yellowing followed by discolouration mainly associated with degradation phenomena, such as oxidation, transformations of the chemical composition, polymer losses, etc. [10,58].

The only exception is provided by titanium white film, exhibiting a ΔE around 4, a small decrease in luminosity (less than 1) and a slight increase in the b* parameter. As observed in studies related to UV ageing, it is possible to hypothesise that, also in this case, the presence of TiO_2_ tends to stabilise the pictorial film [8,59].

- UV ageing: Any relevant morphological change was observed though macro and microscopic observations.

As concerns colourimetric measurements, total colour changes exceeded limit value only for the unpigmented and zinc white films, reaching ΔE values of 13 and 11, respectively. For titanium white and ultramarine blue mock-ups, ΔE is lower than 2. 

This different behaviour may be related to a certain photo-stability demonstrated by TiO_2_ on the acrylic fraction [8,59]. 

For the blue pigment, it can be assumed that the known “whitening” or “greying” effect, also known as “ultramarine disease” [60], entails an overall limited chromatic variation, in the face of even significant chemical transformations (see next paragraph).

In all the mock-ups, the luminosity slightly increases, leading to a slight brightening of the painted surfaces.

Another important finding is the decrease of b* values: Differently to what was observed with high temperature, the UV ageing procedure did not cause the yellowing of the mock-up surfaces.

- High moisture ageing: Under the conditions used, no substantial morphological differences were observed, nor when using UV light. Besides, colour measurements did not register any relevant colour modification in terms of L*a*b*, as reported in Table 4.

#### 3.2.2. Chemical Changes

Through the comparison of the IR spectra of the un-aged and aged mock-ups, the chemical changes are thereinafter presented according to the different ageing procedures:

- Natural ageing
  Any chemical variation was detected for Edelwachs film since IR spectra do not show any relevant modification in their profile. The same consideration can be made for the pigmented mock-ups.
- High temperatures
  Despite appreciable total color variations (see Figure 5 and Table 4), the comparison of the IR spectral profiles between un-aged and HT treated mock-ups evidenced minor modifications of the spectra.

**Figure 6:**
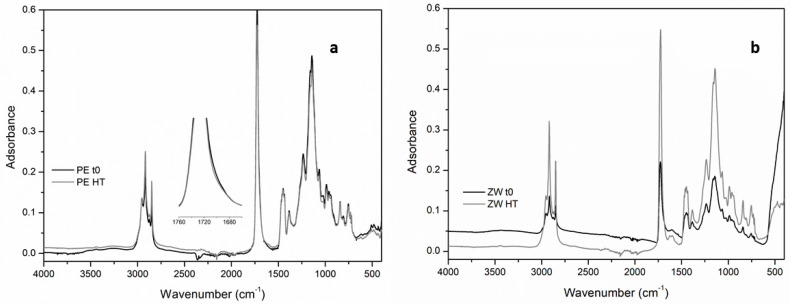
FT-IR-ATR spectra of: (**a**) Edelwachs and (**b**) zinc white mock-ups before ageing (t0) and after high temperature ageing procedure (HT).

In the case of Edelwachs film, whose ΔE and Δb were considerably high, any relevant chemical change was detected. Only a slight broadening of the C=O stretching vibration is visible (Figure 6a), which in literature is associated with the formation of newly photo-degraded products [2].

The zinc white mock-up shows a significant difference in the contribution associated with the pigment (<500 cm^−1^), very evident in the un-aged spectrum (Figure 6b); after HT, this contribution decreases, while the contribution of the organic fraction increases. Furthermore, the presence of a peak at about 1590 cm^−1^ is probably ascribable to the formation of metal carboxylates. Zinc white, as said before, is particularly prone to react with fatty acids and tends to form so-called “zinc soaps”.

The optical observations on this mock-up had highlighted the presence of fluorescent spots, which can be related to these compounds. Due to the small size of the observed spots (less than 10–20 microns—Figure 4), it was not possible to punctually analyse them and confirm their composition.

Similarly, titanium white mock-up shows a general increase of intensity of the organic fraction peaks (particularly, the -CH_3_ stretching). In this case, a broadening of the IR region between 1650–1500 cm^−1^ was registered in the un-aged and aged mock-up, probably due to the pigment-binder interaction. 

In the mock-up containing ultramarine blue, any relevant variation in the IR profiles was detected despite the meaningful colourimetric variations observed.

**Figure 7:**
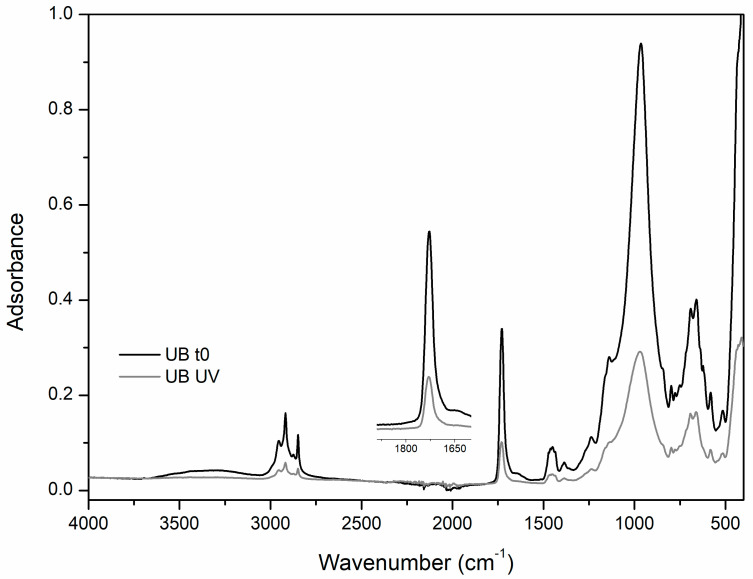
FT-IR-ATR spectra of ultramarine blue mock-ups before ageing (t0) and after UV ageing procedure (UV).

- UV ageing
  In the unpigmented Edelwachs film subjected to UV exposure, the appearance of a small peak at 1773 cm^−1^ was noted, probably indicating the formation of a γ-lactone structure [1,2,3,14].
  Very interesting is in this sample a decrease of the b* parameter (reduction of the yellow component of the color) after the UV exposure, in addition to a general and significant total colour variation. However, the presence of γ-lactones, generally associated with a yellowing of the polymer matrices, in this case was not depicted.
  After 60 days of UV ageing, the mock-ups containing ultramarine blue underwent the most evident degradation processes. In particular, a strong IR intensity decrease of the vibrations referring to the binding media and the contribution of -CH_3_ stretching (2900–2800 cm^−1^), C=O stretching (1726 cm^−1^) e C-O-C peak (Figure 7) were registered. At the same time, a broadening of the C=O peak and the formation of a small peak at 1780 cm^−1^ were registered. As pointed out in similar studies, this variation may be associated with the loss of low molecular weight compounds because of the cross-linking and the fragmentation of side chains [2] and the formation of γ-lactones, as seen for unpigmented Edelwachs film.

This IR spectral modification is consistent with similar studies on ultramarine blue pigment mixed with acrylic-based media after thirty days of UV accelerated ageing. 

However, these spectral variations are not correlated to any significant colourimetric change (See Table 4), since the total colour variation was lower than 3. 

Moreover, for titanium white the color variations with UV were very small, but different to ultramarine blue; the IR pre and post UV spectra are rather superimposable, indicating a fair chemical stability of the film.

In the case of zinc white, despite a ΔE of 5, there are no spectral variations worthy of note.

- HR ageing
  After the ageing procedures using 85% HR, unpigmented and pigmented mock-ups did not show any relevant IR modifications.

#### 3.2.3. Wettability Changes

In the technical datasheet, Edelwachs is described as a “strongly water-repellent” coating wood product. Nevertheless, the contact angle values for the Edelwachs film before ageing procedures were 56 degrees, while for the pigmented mock-ups the Ɵ values slightly decreased (see Table 5).

**Figure 8:**
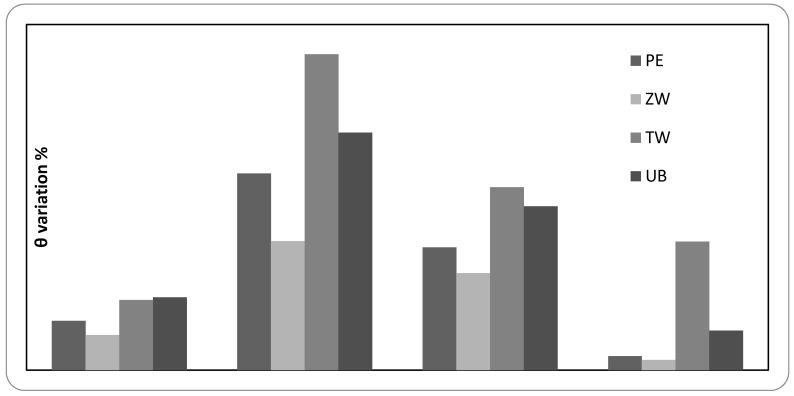
Ɵ variation % values for pigmented and unpigmented mock-ups after natural and accelerated ageing procedures.

After natural and artificial treatments, it was observed that the wettability decreased in all the mock-ups (Figure 8). 

Due to the presence of the acrylic polymer component, a reduction in wettability was expected as a result of the treatments. The data, on the contrary, report a general increase in the contact angle (see Figure 8), in particular in the samples subjected to HT treatment and also in particular for the mock-ups produced by mixing Edelwachs and titanium white, as already observed in other studies [61].

In general, however, the contact angle values do not exceed the threshold of 90°, the limit of non-wettability, the only exception being the titanium white at HT.

The increase in contact angle as a result of artificial ageing may be a consequence of the decrease of polar groups on the surface of the mock-ups [62,63]. Considering also the translucent appearance of the samples even before the treatments, we can reasonably hypothesize the migration of a part of the waxy fraction present in the binder towards the surface with a resulting decrease of its polarity and therefore an increase of the contact angle [57,64]. Such changes were not detected by FT-IR-ATR, which analyses thicknesses in the order of a few microns [65], while it is likely that the waxy fraction has a lower thickness. 

The formation of degradation products of the acrylic fraction, detected on Edelwachs and ultramarine blue after UV ageing and on Edelwachs after HT, in addition to the possible presence of metal soaps in zinc white mock-ups, should have increased the wettability of the painted surfaces. Since this increase was not registered, we can assume that there is a minimum quantitative threshold below which the presence of these polar groups does not affect wettability. 

An increase in contact angle could also be due to an increase in roughness, although this relationship appears to be significant for contact angles above 90° and roughness up to 0.1 μm. Given these considerations, it is believed that the decrease in wettability is related to a physical rather than a chemical process.

#### 3.2.4. General Considerations on the Effects of the Ageing Procedures 

Table 6 provides an overview of the results obtained by OM, colourimetric measurements, FT-IR-ATR and contact angles measurements.

In general, unlike what would be expected from acrylic-based binding media, the effects produced by ageing procedures are not evident, except for the colour variations (ΔE and b* in particular) in mock-ups subjected to UV and HT and the increase of contact angles in all cases.

In conclusion, Table 6 shows that Edelwachs and the mock-ups subjected to UV and HT ageing gave the most evident overall variations.

### 3.3. Evaluation of the Thermal Behaviour

**Figure 9:**
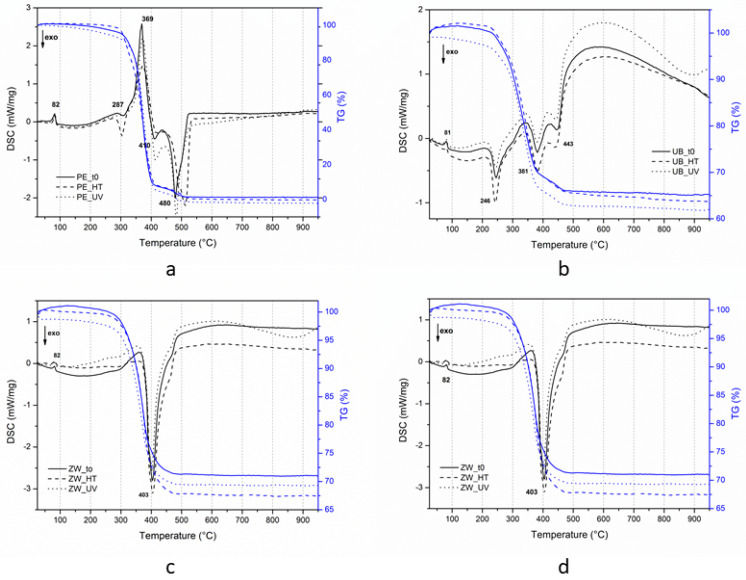
TG-DSC curves of un0aged (t0), high temperature (HT) and UV aged mock-ups: (**a**) Edelwachs (PE); (**b**) ultramarine blue (UB); (**c**) titanium white (TW); (**d**) zinc white (ZW).

Considering the results presented in Table 6, the un-aged mock-ups (at t0) and those treated with UV and HT were analyzed via TG-DSC and the corresponding TG-DSC curves are depicted in Figure 9a–d.

The thermogramm of the un-aged Edelwachs film is characterised by a mass loss in the 30–300 °C range associated with the loss of volatile components and low molecular weight compounds [3,15] and two other mass losses between 300–400 and 400–500 (1000) °C. Edelwachs loses the whole weight (99.8%) within 500 °C (Table 7).

Regarding the pigmented mock-ups, the total weight loss reflects the PVC of the inorganic pigment mixed to the binder. The values were about 38% for ultramarine blue, between 45 and 48% for titanium white and between 31 and 39% for zinc white. The weight variations may be due to small differences in the making of the painted films, more than the influence of the accelerated ageing procedures.

Based on the DSC curve of Edelwachs at t0 (Figure 9a), some considerations can be made [3,66,67,68,69]:

- The glass transition temperature (Tg) occurs at about 36 °C: This value is probably due to the influence of the wax components mixed with acrylic monomers.
- The endothermic process at 82 °C refers to a solid–liquid phase change (melting) of the waxy components, where no mass variations are involved.
- In the range of 300–500 °C, an endothermic (369 °C) and two exothermic processes (410 and 480 °C) were registered: The first is due to the melting of the acrylates and the two remaining exothermic peaks to their decomposition (combustion). Generally, the position of these decomposition processes may shift depending on the monomers involved: In this case, they depend on the overall contributions of the four monomers (MA, MMA, BMA and nBA), identified by Py-GC-MS.
- At 287 °C, it is also possible to detect a not well resolved exothermic peak, likely associated with the decomposition of the waxy components.

The DSC curve of the Edelwachs film after UV ageing shows a thermal profile similar to the one at t0 (Figure 9a). Differently from what was observed in the literature, the UV exposure does not seem to have a strong influence on the thermal stability of the acrylic fraction: This corroborates the IR observations about chemical changes. 

The behaviour is different after high temperature ageing (Figure 9a). At about 300 °C, a well-defined exothermic peak is depicted, referring to the decomposition of the waxy components [67].

A shift of the peak around 500 °C was also observed: This may suggest an increased thermal stability of the polymeric network, despite any relevant chemical changes (as highlighted by FT-IR-ATR).

**Figure 10:**
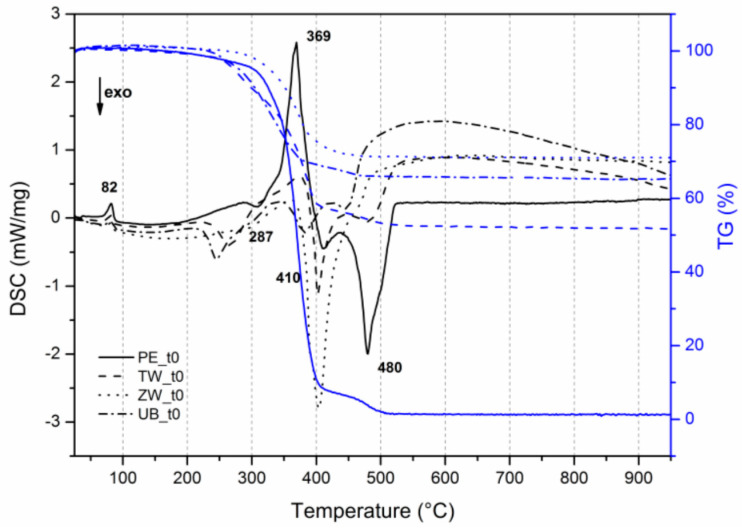
TG-DSC profiles of unpigmented and pigmented mock-ups at t0.

The DSC curves show that the presence of pigment in the mock-ups, even before the ageing procedures, produces a difference between the pigmented and unpigmented films (Figure 10). As expected, the addition of a pigment does change the thermal profile of the pure polymer [3].

Concerning the Tg, it is interesting to notice that the Tg values are rather constant, except for ultramarine blue mock-ups (Table 7), which values vary from 36/38 °C to 29/31 °C. 

As observed in the unpigmented mock-ups, the DSC curves of aged pigmented mock-ups did not show any significant modifications if compared with the un-aged ones (Figure 9b–d). This finding is in contrast with previous research where the UV and HT ageing treatments resulted in affecting the thermal stability of the mixed pigment-acrylic binder.

The differences that we find between the painted layers produced with the different pigments concern, in particular, the main acrylic exothermic peaks (between 400 and 500 °C), shifted at a lower temperature in the case of titanium white and ultramarine blue, probably indicating that the acrylic fraction of the polymer is less thermally stable in these pigmented films. 

The case of the paint film obtained with zinc white is different, where the thermal profile is completely different from pure Edelwachs and the other two pigments. Apart from the endothermic peak at about 80 °C, in zinc white mock-ups both the peaks at about 280 and 370 °C are no longer visible, indicating a possible interaction between the components of the complex paint mixture. The main acrylic exothermic peaks (between 400 and 500 °C) are reduced to a distinctive intense peak centered around 400 °C, shifted at a lower temperature. This could correspond to the final combustion of the acrylic fraction, while the small shoulder at 390 °C may be related to its initial decomposition process. According to the literature, the presence of ZnO generally should have a stabilizing effect on acrylic polymers [70]. In our case, the presence of ZnO leads to a delay of the melting process and an advance of the combustion temperature of the acrylic fractions. This different behaviour may be attributed to the high PVC of ZnO in the mock-ups (32%) compared to literature studies.

After the HT procedure, a different thermal profile was observed in this range. This could be due to processes observed by microscopy and recorded by FT-IR-ATR, likely associated with acrylic/wax phase separation, additives migration and/or metal carboxylates formation.

## 4. Conclusions

The paper focuses on evaluating the chemical and thermal stability under accelerated ageing of Edelwachs, a commercial product composed of a water-based emulsion of different acrylic polymers (MA, MMA, nBA, nBMA), Carnauba and microcrystalline waxes and various additives. Edelwachs, produced for wood coating, was unconventionally used as a paint binder by contemporary artists, such as Fabio Nones and his pupils

This study highlighted the good chemical stability of Edelwachs, even when subjected to accelerated ageing procedures. The selected ageing conditions (natural ageing in laboratory, high temperature, high relative humidity and photo-oxidation through UV light) did not strongly affect the chemical composition of the polymers present in the complex commercial formulation.

The naturally aged film paints revealed low variations in terms of L*, a*, b* with ΔE values lower than 1 for pigmented mock-ups and 3 for the unpigmented Edelwachs films, associated with a negative variation of the b* parameter (yellowing).

High temperature ageing produced, for all the mock-ups, the most relevant colourimetric variations. UV ageing, which in the literature is reported to be the most impactful on the acrylic components, was significant but not relevant as expected. Only the unpigmented and zinc white mock-ups showed relevant ΔE variations, reaching values of 13 and 11, for UV aging, and of 37 and 11 for HT ageing.

The lack of a direct correlation between chemical transformations detectable by FT-IR-ATR and the observed colourimetric variations, specifically for the parameter b* (yellowing), is significant. The b*parameter is generally associated with the formation of chromophore groups, which have not been detected in the current study except in some cases and in a very limited amount. In particular, the formation of chromophore groups may find a confirmation in the case of Edelwachs film subjected to high temperature, where a slight broadening of the C=O stretching vibration was detected by FT-IR-ATR.

Despite the meaningful colourimetric variations observed after high temperature ageing, ultramarine blue films did not highlight any relevant variation in the FT-IR-ATR spectra.

In the case of the mock-ups containing zinc white, it was possible to appreciate the influence of the pigment thanks to the presence of a peak at about 1590 cm^−1^ in the FT-IR-ATR spectrum after high temperature ageing. This is likely ascribable to the formation of metal carboxylates, as also observed in many contemporary paintings containing this white pigment.

The thermal behavior, observed by TG-DSC, underlined the influence of the pigments on the acrylic polymeric fraction. The DSC profile of the pigmented mock-ups containing zinc white is peculiar, where the main acrylic exothermic peaks between 400 and 500 °C are reduced to a distinctive intense peak at around 400 °C. According to the literature, the presence of ZnO generally has a stabilising effect on acrylic polymers. In our case, this is not evident probably due to the high PVC of ZnO pigment in the mixtures (about 32%), which leads to a delay in the melting process and an advance of the combustion temperature.

The findings obtained in terms of wettability (contact angle measures) were very interesting. Wettability underwent a general and significant decrease, both in the case of unpigmented and pigmented Edelwachs mock-ups. This behaviour may be related to a physical rather than a chemical process, due to a partial migration of the waxy fraction present in the binder, and a consequent decrease of the polar groups on the surface of the mock-ups.

Wettability data, associated with chromatic variations and chemical behaviour, suggested that there is not always a direct relationship between these characteristics, in particular for complex mixtures containing waxy components. They can be considered, in fact, as a “protective” for the acrylic fraction and, at the same time, they can reduce the film wettability.

The use of binders not intentionally formulated for painting purposes is quite common among contemporary artists, and, based on the collected data, Edelwachs proves to be suitable for artistic purposes. As observed, the addition of waxes in the formulation seems to improve its stability.

It would be interesting to expand and study the effect of waxes on different water-based painting binders, such as vinyl-based formulations, in terms of stability over time. The addition of waxes to polymeric mixtures may help in reducing degradation processes and therefore increase the durability of works of art, even in conservation environments where microclimatic control is not feasible. The sensitivity of paint films presenting waxy components needs, however, to be evaluated towards conservation interventions such as cleaning procedures.

## Figures and Tables

**Table 1:** List of mock-ups, description, pigment volume concentration (PVC) and dried film thickness.

Mock-Ups	Description	PVC (%) *	Film Thickness (μm)
PE	Edelwachs	-	190 ± 10
TW	Edelwachs + Titanum White	25%	170 ± 13
ZW	Edelwachs + Zinc White	32%	289 ± 44
UB	Edelwachs + Ultramarine Blue	31%	220 ± 13

* PVC—pigment volume concentration—(V_pigments_/V_pigments_ + V_Binder_) × 100%.

**Table 2:** XRF and Fourier Transformed Infrared Attenuated Total Reflectance (FT-IR-ATR) results on the powdered pigments.

Name	XRF Results *	FT-IR-ATR Results	Comments
TW	Ti +++	481 cm^−1^ TiO_2_	Titanium Oxide
ZW	Zn +++, Fe (tr)	500–400 cm^−1^ oxides	Zinc Oxide
UB	S ++, Si +, Al +, Ti +, Fe +, Na (tr), Ca (tr)	998 cm^−1^ Si-O-Si 443 cm^−1^ Si-O-Si	Ultramarine Blue with traces due to natural impurities

* +++ = very abundant, ++ = abundant, + = present, (tr) = traces.

**Table 3:** Main compounds and their molecular ion and retention time (RT) values detected through Py-GC-MS analysis of Edelwachs.

RT (min) (min)	** M+	COMPOUND	RT (min) (min)	** M+	COMPOUND
0.94	56 (41)	2 butene	9.34	242 (55)	1-Hexadecanol
1.29	72 (44)	Butanal	10.56	244 (115)	Glutaric acid*
1.38	86 (55)	**Methyl Acrylate**	10.81	256 (57)	1-Hexadecanol, 2-methyl-
1.46	88 (29)	Methyl propionate	10.88	239 (127)	Pyrrolidine, 1-(7-oxo-2,4,6-trimethylheptanoyl)-
1.69	74 (56)	1-butanol	10.92	536 (43)	1-Heptatriacotanol
1.80	102 (43)	Propanoic acid, 2-methyl- *	11.44	252 (82)	13-Heptadecyn-1-ol
1.99	100 (41)	**Methyl methacrylate**	11.49	266 (55)	9-Nonadecene
2.70	112 (43)	1-octene	12.14	256 (57)	1-Hexadecanol, 2-methyl-
2.82	114 (55)	Butanoic acid, 2-methylene- *	12.33	270 (74)	Hexadecanoic acid *
2.95	116 (43)	**Acetic acid, butyl ester**	12.61	278 (79)	10-Heptadecen-8-ynoic acid (E)- *
3.05	86 (71)	2-Buten-1-ol, 2-methyl-	12.76	256 (57)	1-Hexadecanol, 2-methyl-
3.38	128 (41)	Butanoic acid, 3-methyl-2-methylene- *	13.39	314 (41)	4-Methoxycarbonylmethylundec-3-enedioic acid *
3.84	128 (55)	***n*-Butyl Acrylate**	13.43	296 (55)	trans-13-Octadecenoic acid *
3.94	130 (57)	Propanoic acid *	13.56	314 (41)	4-Methoxycarbonylmethylundec-3-enedioic acid *
4.25	142 (83)	4-Pentenoic acid, 2,4-dimethyl- *	13.60	298 (74)	Heptadecanoic acid, 16-methyl- *
4.55	126 (67)	4-Pentenoic acid, 2-methylene- *	14.04	472 (57)	Docosyl pentafluoropropionate
4.70	142 (41)	***n*-Butyl methacrylate**	14.15	298 (74)	Octadecanoic acid *
4.94	148 (49)	2-Propanol, 1-(2-methoxy-1-methylethoxy)-	14.71	490 (57)	17-Pentatriacontene
5.05	148 (59)	2-Propanol, 1-(2-methoxypropoxy)-	14.94	326 (74)	Eicosanoic acid *
5.58	156 (83)	*n*-Butyl tiglate	15.13	N/A (134)	**trimer *n*-butyl-acrylate**
5.84	210 (41)	E-11,13-Tetradecadien-1-ol	16.12	366 (43)	Octadecane, 3-ethyl-5-(2-ethylbutyl)-
6.39	170 (97)	Cyclopentanecarboxylic acid *	17.48	604 (57)	Tritetracontane
6.42	122 (122)	Phenol, 2,5-dimethyl-	17.69	382 (74)	Tetracosanoic acid *
6.80	168 (55)	Cyclopentane, 1-methyl-2-(4-methylpentyl)-, trans-	18.12	490 (57)	17-Pentatriacontene
6.97	170 (77)	2,4-Octadienoic acid, 7-hydroxy-*[R-(E, E)]-	18.78	490 (57)	17-Pentatriacontene
7.26	290 (74)	13,16-Octadecadiynoic acid *	18.80	618 (57)	Tetratetracontane
7.38	186 (81)	2-Carboxymethyl-3-methyl-cyclopentanecarboxylic acid	19.01	410 (74)	Hexacosanoic acid *
7.69	182 (43)	1-Tridecene	19.43	N/A (57)	C32 alcohol, methoxy Carnaubawax
7.90	278 (79)	10-Heptadecen-8-ynoic acid (E)- *	19.72	504 (55)	9-Hexadecenoic acid, 9-octadecenyl ester, (Z, Z)-
8.09	224 (74)	12-Tridecynoic acid *	20.07	604 (57)	Tritetracontane
8.16	154 (95)	2-Octynoic acid *	20.28	438 (74)	Octacosanoic acid *
8.47	294 (41)	8-Octadecynoic acid *	20.69	612 (57)	Dotriacontyl pentafluoropropionate
8.54	242 (55)	1-Hexadecanol	21.86	490 (57)	17-Pentatriacontene
8.85	174 (59)	Butanedioic acid, 2,3-dimethyl- *	22.18	594 (82)	Tetracontane-1,40-diol
9.04	200 (43)	4-Pentenoic acid, 4-methoxycarbonyl- *	22.58	N/A (57)	C32 alcohol, methoxy Carnaubawax
9.28	294 (81)	10-Octadecynoic acid *			

* as methyl esters, ** in brackets the most abundant fragment.

**Table 4:** Colour changes calculated as ΔE, ΔL*, Δa* and Δb* after natural and accelerated ageing.

Mock-Ups		Colour Changes (L*a*b*)			
		ΔE	ΔL*	Δa*	Δb*
**PE**	PEn.ag	2.66	0.55	0.10	−2.60
	PEHT	36.84	−15.18	12.09	31.31
	PEUV	12.85	2.14	1.27	−12.61
	PEHR	3.63	3.34	0.79	1.20
**ZW**	ZWn.ag.	0.67	−0.10	0.06	0.66
	ZWHT	10.62	−4.79	1.22	9.39
	ZWUV	5.05	1.18	0.30	−4.90
	ZWHR	0.13	0.03	−0.09	−0.10
**TW**	TWn.ag.	0.45	−0.25	0.08	−0.36
	TWHT	3.57	−0.90	0.24	3.45
	TWUV	1.66	0.69	0.23	−1.49
	TWHR	0.14	0.05	0.07	−0.11
**UB**	UBn.ag.	0.77	−0.53	0.17	0.53
	UBHT	15.25	−7.22	−5.19	12.39
	UBUV	1.58	0.97	−0.01	−1.25
	UBHR	1.21	−0.85	0.31	0.81

**Table 5:** Contact angle measurements of the mock-ups before and after natural and accelerated ageing procedures.

Mock-Ups		Contact Angles (θ)
**PE**	PE (t0)	56.16
	PEn.ag	64.15
	PEHT	88.09
	PEUV	76.11
	PEHR	50.79
**ZW**	ZW (t0)	51.09
	ZWn.ag.	67.26
	ZWHT	83.84
	ZWUV	78.19
	ZWHR	62.88
**TW**	TW(t0)	48.94
	TWn.ag.	58.88
	TWHT	93.67
	TWUV	74.54
	TWHR	67.15
**UB**	UB (t0)	51.09
	UBn.ag.	61.85
	UBHT	86.18
	UBUV	75.28
	UBHR	56.91

**Table 6:** Overview of the overall effects of natural and accelerated ageing procedures on mock-ups.

	Mock-Ups	Ageing Procedures			
		Natural Ageing	High Temperatures	UV Irradiation	85% RH
**Morphological variations**	PE	-	-	-	-
	TW	-	+	-	-
	ZW	+	+	+	-
	UB	-	-	-	-
**Colour variations** **(DE > 5)**	PE	-	+	+	-
	TW	-	-	-	-
	ZW	-	+	+	-
	UB	-	+	-	-
**Chemical variations** **(FT-IR-ATR)**	PE	-	-	-/+	-
	TW	-	+/-	-/+	-
	ZW	-	+/-	-	-
	UB	-	-	+/-	-
**Wettability** **(Ɵ)**	PE	>	>	>	>
	TW	>	>>	>>	>>
	ZW	>	>	>	>
	UB	>	>>	>>	>

+ = relevant registered variation, +/- = visible variation, -/+ = slightly visible, - = not registered, > = increase, >> = high increase.

**Table 7:** Total weight loss % and glass transition temperature (Tg) of the un-aged and aged mock-ups.

Mock-Ups		Total Weight Loss %	Glass Transition (Tg)
**PE**	T_0_	99.8	36 °C
	UV	99.9	36 °C
	HT	99.9	38 °C
**UB**	T_0_	37.0	30 °C
	UV	39.9	29 °C
	HT	37.1	31 °C
**TW**	T_0_	49.3	36 °C
	UV	48.8	38 °C
	HT	44.9	38 °C
**ZW**	T_0_	39.1	36 °C
	UV	31.3	38 °C
	HT	37.1	36 °C
